# Assessment of the Diagnostic Accuracy of CT as Compared to MRI in Detecting Metastases in Patients With Colorectal Cancer

**DOI:** 10.7759/cureus.66125

**Published:** 2024-08-04

**Authors:** Qaed S Alhammami

**Affiliations:** 1 Radiology, Najran University, Najran, SAU

**Keywords:** comparison, diagnostic accuracy, magnetic resonance imaging, computed tomography, metastases, colorectal cancer

## Abstract

This study aimed to compare the diagnostic accuracy of computed tomography (CT) and magnetic resonance imaging (MRI) in detecting metastases of colorectal cancer (CRC) in a hospital in Najran, Saudi Arabia. A total of 51 patients with CRC were included in the study. The radiological findings of metastatic lesions and the diagnostic accuracy measures of CT compared to MRI were analyzed. The results showed that CT had a false negative rate of 7.8%, a false positive rate of 7.8%, a true negative rate of 27.5%, and a true positive rate of 56.9% in detecting metastases. Diagnostic accuracy measures varied based on the number of metastatic lesions, with higher sensitivity observed for cases with fewer lesions. Gender, timing of imaging in relation to surgical intervention, and administration of nonsurgical therapy showed significant associations with diagnosis mismatch between CT and MRI. The site of metastases and the site of the primary tumor in the colon also demonstrated significant associations with diagnosis mismatch. The size of the largest metastasis detected by MRI was significantly associated with diagnosis mismatch. The overall diagnostic accuracy of CT in detecting any metastases, compared to MRI as the reference standard, was estimated to have a sensitivity of 87.8%, a specificity of 77.8%, a positive predictive value of 87.8%, and a negative predictive value of 77.8%. This study provides valuable insights into the comparative diagnostic performance of CT and MRI in detecting metastases of CRC, highlighting the importance of considering patient characteristics, disease outcome, and tumor characteristics in the interpretation of imaging results.

## Introduction

Colorectal cancer (CRC) is one of the most common malignancies worldwide, and its incidence is increasing globally. The presence of metastases in patients with CRC has significant implications for prognosis and treatment decisions. Therefore, accurate detection of metastases is essential in the management of patients with CRC [1,2].

Computed tomography (CT) and magnetic resonance imaging (MRI) are two imaging modalities commonly used in the evaluation of patients with CRC. CT has been widely used for the detection of metastases due to its high spatial resolution and widespread availability. However, MRI has the advantage of superior soft tissue contrast, which can improve the detection of small metastases and the evaluation of peritumoral tissue [3-5].

Several studies have compared the accuracy of CT and MRI scans in detecting colon cancer metastases. One study found that MRI scans may be better at detecting the metastasis of CRC, while another study found no significant difference in sensitivity between whole-body MRI and standard pathways (which may include CT scans) [6-9]. However, in terms of detecting T3-4 disease specifically, MRI was found to have a better performance than CT, with higher specificity and area under the curve (AUC) [10].

A study specifically comparing MRI scans and CT scans in bowel cancer found that they had identical accuracy in detecting liver metastases but CT had slightly higher specificity while MRI was more sensitive [11]. Another study found that CT during arterial portography had the highest detection rate for hepatic metastases, with an overall detection rate of 81-94% [12].

Overall, the choice between CT and MRI for detecting colon cancer metastases may depend on the specific situation and factors such as the location and size of the suspected metastases, patient preference, and availability of resources. It is important to confer with a healthcare provider to determine the most appropriate imaging modality for each individual case.

This study's aim is to provide highlights of the diagnostic performance of CT and MRI in the detection of metastases in patients with CRC, which can assist clinicians in selecting the appropriate imaging modality for the detection of metastases in these patients. The specific objectives are to provide a comprehensive evaluation of the diagnostic accuracy of CT versus MRI and compare their performance in detecting metastases. The study compared the diagnostic performance of CT with that of MRI in detecting metastases in patients with CRC in a single center in Najran, Saudi Arabia.

## Materials and methods

This was a retrospective observational study conducted at King Khalid Hospital, Najran, Saudi Arabia, from January 1, 2020, to December 31, 2022, to examine and compare the diagnostic accuracy of CT and MRI in the detection of metastases related to CRC. The study was approved by the Ethics Committee Najran University, Najran (approval number: 202312-076-016106-037171). Patient data were anonymized to protect confidentiality and handled in accordance with the ethical guidelines governing medical research.

Study participants

A cohort of 51 individuals who had been diagnosed with CRC and had undergone CT and MRI scans for the purpose of assessing metastases at King Khalid Hospital during the study period were enrolled in the study. The participants encompassed both male and female patients spanning various age groups and having diverse comorbidity profiles. Patients who had previously received treatment for metastases or had undergone CT or MRI scans at a different medical institution were excluded from the study.

Data collection

Patient data were meticulously gathered from medical records, encompassing a wide range of information such as demographic details, comorbidity history, previous neoplasms, timing of imaging in relation to surgical procedures, and the use of nonsurgical therapies. Furthermore, specific details regarding the primary tumor site in the colon, TNM (Tumour, Node, Metastasis) stage of colon cancer, histological type, tumor differentiation grade, presence of metastases, and the outcomes of CRC were meticulously recorded.

Imaging procedure

The imaging procedures involved both CT and MRI scans for the patients under study. CT scans were performed following standard protocols, and the resulting images were thoroughly reviewed by seasoned radiologists. Similarly, MRI scans were conducted using appropriate sequences, and the radiologists meticulously examined the images for the presence of any metastases.​​​​​​​​​​​​​​

The study involved the utilization of a GE 1.5T MRI system (General Electric Company, Boston, Massachusetts, United States) for oncology imaging and a GE 128 slice CT scanner for CRC staging. The MRI protocol included various sequences such as axial single-shot fast spin-echo (SS-FSE), coronal SS-FSE, axial T2 fat-suppressed (FS), axial T1 (in/out phase), axial diffusion-weighted imaging (DWI), axial three-dimensional (3D) T1 FS pre and post-contrast injection (late arterial and portovenous delay phases), and coronal 3D T1 FS post-contrast injection. The contrast agent used was Dotarem, with a dosage of 0.2 mL/kg for patients with a glomerular filtration rate (GFR) greater than or equal to 60, and 0.1 mL/kg for those with a GFR less than 50, administered at a rate of 2 mL/sec.

On the other hand, the CT protocol for CRC staging focused on the CAP (chest, abdomen, and pelvis) region and utilized an injection cannula size of 20G with a flow rate of three seconds. The oral and rectal contrast agents used were water, while the IV contrast agent was Omnipaque 350. The scan included phases with a scan delay of 70 seconds. These detailed protocols were followed meticulously to ensure comprehensive and high-quality imaging for accurate diagnosis and staging of oncological conditions.

Data analysis

The collected data underwent thorough analysis utilizing a variety of statistical methods. Descriptive statistics, including frequencies and percentages, were employed to provide a comprehensive summary of patient characteristics, disease outcomes, and radiological findings. Diagnostic accuracy metrics such as sensitivity, specificity, positive predictive value, and negative predictive value were computed to compare the performance of CT with MRI. Furthermore, the relationship between patient characteristics, disease outcomes, and discrepancies in metastasis diagnosis between CT and MRI was examined through chi-square tests. Statistical significance was established at a p-value of less than 0.05.

## Results

A total of 51 patients with CRC were included in the study. The sample characteristics, history, and CRC diagnosis are presented in Table 1*.* Among the 51 participants, 43.1% were aged 22-50 years, while 56.9% were aged 51-69 years. The gender distribution was almost equal, with 43.1% females and 56.9% males. Approximately half of the participants had comorbidities (49%), while the rest did not. Most patients (90.2%) did not have a history of other neoplasms besides CRC. Imaging was performed both before and after surgical intervention in a similar proportion (49% and 51%, respectively). The majority of patients were classified as stage IV CRC (62.7%), and 68.6% of patients received nonsurgical therapy. The most common site of metastases was the liver (39.2%). The outcome of CRC showed that 56.9% of patients were alive with metastatic disease, 35.3% were alive without metastatic disease, and 7.8% had passed away. The final diagnosis revealed that 64.7% of patients had metastatic colon cancer while 35.3% had non-metastatic colon cancer. The site of the primary tumor in the colon varied, with the rectum being the most common location (41.2%), followed by the sigmoid colon (29.4%), descending colon (15.7%), ascending colon (9.8%), and transverse colon (3.9%). The TNM classification of the primary tumor indicated that the majority of patients had T3 tumors (62.7%), followed by T4 (29.4%), T2 (5.9%), and T1 (2%). Adenocarcinoma was the most common histological type (90.2%), while mucinous adenocarcinoma (7.8%) and signet ring cell carcinoma (2%) were less frequent. In terms of tumor differentiation grade, most tumors were moderately differentiated (70.6%) while only 2% were well differentiated.

**Table 1:** Characteristics of the sample, history, and CRC diagnosis (n=51). CRC: colorectal cancer TNM Classification: T describes the size of the tumor and any spread of cancer into nearby tissue; N describes spread of cancer to nearby lymph nodes; and M describes metastasis (spread of cancer to other parts of the body).

Parameter		Frequency (Percentage)
Age, years	22 - 50	22 (43.1%)
	51 - 69	29 (56.9%)
Gender	Female	22 (43.1%)
	Male	29 (56.9%)
Comorbidities	No	26 (51%)
	Yes	25 (49%)
History of other neoplasms than CRC	No	46 (90.2%)
	Yes	5 (9.8%)
Imaging performed before or after the surgical intervention	After	25 (49%)
	Before	26 (51%)
Staging of the CRC	Stage I	1 (2%)
	Stage II	3 (5.9%)
	Stage III	15 (29.4%)
	Stage IV	32 (62.7%)
Nonsurgical therapy recieved	No	16 (31.4%)
	Yes	35 (68.6%)
Site of metastases	Liver	20 (39.2%)
	None	16 (31.4%)
	Other	15 (29.4%)
Outcome of the CRC	Alive with metastatic disease	29 (56.9%)
	Alive without metastatic disease	18 (35.3%)
	Dead	4 (7.8%)
Final diagnosis of the patient	Metastatic colon cancer	33 (64.7%)
	Non-metastatic colon cancer	18 (35.3%)
Site of the primary tumor in the colon	Ascending colon	5 (9.8%)
	Descending colon	8 (15.7%)
	Rectum	21 (41.2%)
	Sigmoid colon	15 (29.4%)
	Transverse colon	2 (3.9%)
TNM classification of the primary tumor at the time of diagnosis	T1‎	1 (2%)
	T2‎	3 (5.9%)
	T3‎	32 (62.7%)
	T4‎	15 (29.4%)
Histological type of colon cancer	Adenocarcinoma	46 (90.2%)
	Mucinous adenocarcinoma	4 (7.8%)
	Signet ring cell carcinoma	1 (2%)
Differentiation grade of the tumor	Moderately differentiated	36 (70.6%)
	Not specified	10 (19.6%)
	Poorly differentiated	4 (7.8%)
	Well differentiated	1 (2%)

Table 2presents the radiological findings of metastatic lesions and the diagnostic accuracy of CT compared to MRI in the detection of metastases in patients with CRC. In terms of the size of the largest metastasis detected by MRI, the majority of cases (47.1%) had metastases measuring 1-2 cm, followed by 9.8% with metastases measuring 2-3 cm. A smaller percentage of cases had metastases greater than 3 cm (3.9%), less than 1 cm (3.9%), or no detectable metastases (35.3%). Regarding the number of metastatic lesions detected, CT identified one metastatic lesion in 19.6% of cases, two lesions in 31.4% of cases, three lesions in 7.8% of cases, and four or more lesions in 5.9% of cases. In comparison, MRI detected one metastatic lesion in 9.8% of cases, two lesions in 9.8% of cases, three lesions in 19.6% of cases, four or more lesions in 3.9% of cases, and four or more lesions in 21.6% of cases. Notably, both imaging modalities identified no metastatic lesions in 35.3% of cases. The diagnostic accuracy measures provide insights into the performance of CT and MRI in detecting metastases. Overall, the diagnostic accuracy of metastasis detection was assessed using the metrics of false negatives (FNs), false positives (FPs), true negatives (TNs), and true positives (TPs). In the entire cohort (n=51), CT had an FN rate of 7.8%, FP rate of 7.8%, TN rate of 27.5%, and TP rate of 56.9%. Furthermore, the diagnostic accuracy for specific scenarios was evaluated. For patients without metastases (n=13), CT and MRI showed perfect specificity with no FN or FP cases. In cases with one metastasis (n=9), there were no FN cases, but CT had an FP rate of 11.11% and a TP rate of 55.56%. In the detection of two metastases (n=8), CT did not produce any FP cases, had an FN rate of 25%, and a TP rate of 50%. Similarly, in cases with three metastases (n=7), CT had no FP cases, an FN rate of 28.57%, and a TP rate of 71.43%. Finally, for cases with four or more metastases (n=14), CT had no FP cases, an FN rate of 21.43%, and a TP rate of 78.57%.

**Table 2:** Radiological findings of metastatic lesion and diagnostic accuracy of CT with MRI as a reference (n=51). MRI: magnetic resonance imaging; CT: computed tomography

Parameter		Frequency (Percentage)
Size of the largest metastasis detected by MRI	1-2 cm	24 (47.1%)
	2-3 cm	5 (9.8%)
	Greater than 3 cm	2 (3.9%)
	Less than 1 cm	2 (3.9%)
	None	18 (35.3%)
Number of metastatic lesions detected by CT	One	10 (19.6%)
	Two	16 (31.4%)
	Three	4 (7.8%)
	Four or more	3 (5.9%)
	None	18 (35.3%)
Number of metastatic lesions detected by MRI	One	5 (9.8%)
	Two	5 (9.8%)
	Three	10 (19.6%)
	Four or more	2 (3.9%)
	‎4 or more‎	11 (21.6%)
	None	18 (35.3%)
Diagnostic accuracy (overall metastasis detection)	FN	4 (7.8%)
	FP	4 (7.8%)
	TN	14 (27.5%)
	TP	29 (56.9%)
Diagnostic accuracy in detection of no metastases (n=13)	FN	0 (0%)
	FP	0 (0%)
	TN	13 (100%)
	TP	0 (0%)
Diagnostic accuracy in detection of one metastasis (n=9)	FN	0 (0%)
	FP	1 (11.11%)
	TN	3 (33.33%)
	TP	5 (55.56%)
Diagnostic accuracy in detection of two metastases (n=8)	FN	2 (25%)
	FP	0 (0%)
	TN	2 (25%)
	TP	4 (50%)
Diagnostic accuracy in detection of three metastases (n=7)	FN	2 (28.57%)
	FP	0 (0%)
	TN	0 (0%)
	TP	5 (71.43%)
Diagnostic accuracy in detection of four metastases or more (n=14)	FN	3 (21.43%)
	FP	0 (0%)
	TN	0 (0%)
	TP	11 (78.57%)

Table 3provides an analysis of the association between patient characteristics, disease outcome, and the presence of metastasis diagnosis mismatches between CT and MRI in the cohort of 51 patients. The table presents the frequency of matches and mismatches, as well as the chi-square (X^2^) statistic and p-values for each parameter. Age was evaluated as a potential factor influencing the mismatch between CT and MRI diagnoses. Among patients aged 22-50 years, 45.5% had a match in diagnoses, while 54.5% experienced a mismatch. Similarly, for patients aged 51-69 years, 51.7% had a match and 48.3% had a mismatch. However, the chi-square test did not indicate a significant association between age and diagnosis mismatch (X^2^ = 0.197, p = 0.657). Gender was found to be significantly associated with diagnosis mismatch. Among female patients, 72.7% had a match in diagnoses while 27.3% experienced a mismatch. In contrast, among male patients, only 31% had a match, and a significant majority of 69% had a mismatch (X^2^ = 8.702, p = 0.003). The presence of comorbidities and a history of neoplasms other than CRC did not show a significant association with diagnosis mismatch. When considering comorbidities, 57.7% of patients without comorbidities had a match, compared to 40% of patients with comorbidities (X^2^ = 1.596, p = 0.206). For patients without a history of neoplasms other than CRC, 47.8% had a match while 52.2% had a mismatch. Among patients with a history of other neoplasms, 60% had a match and 40% had a mismatch (X^2^ = 0.267, p = 0.605). The timing of imaging in relation to surgical intervention was significantly associated with diagnosis mismatch. Among patients who underwent imaging after surgical intervention, only 24% had a match, while a substantial majority of 76% experienced a mismatch. In contrast, when imaging was performed before surgical intervention, 73.1% of patients had a match, and 26.9% had a mismatch (X^2^ = 12.284, p = 0.000). The administration of nonsurgical therapy showed a significant association with diagnosis mismatch. Among patients who did not receive nonsurgical therapy, a higher proportion of 75% had a match in diagnoses, compared to 37.1% of patients who received nonsurgical therapy (X^2^ = 6.297, p = 0.012). Disease outcome, as indicated by the CRC outcome and the patient's final diagnosis, demonstrated significant associations with diagnosis mismatch. Among patients who were alive with metastatic disease, 27.6% had a match while 72.4% had a mismatch. For patients alive without metastatic disease, a substantial majority of 77.8% had a match and only 22.2% had a mismatch. Among deceased patients, 75% had a match and 25% had a mismatch in diagnoses (CRC outcome: X^2^ = 12.368, p = 0.002; final diagnosis: X^2^ = 9.206, p = 0.002).

**Table 3:** Metastasis diagnosis mismatch in association with patient characteristics and disease outcome (n=51). CRC: colorectal cancer

Parameter		CT versus MRI metastasis diagnosis		X^2^	P-value
		Match	Mismatch		
Age, year	22 - 50	10 (45.5%)	12 (54.5%)	0.197	0.657
	51 - 69	15 (51.7%)	14 (48.3%)		
Gender	Female	16 (72.7%)	6 (27.3%)	8.702	0.003
	Male	9 (31%)	20 (69%)		
Comorbidities	No	15 (57.7%)	11 (42.3%)	1.596	0.206
	Yes	10 (40%)	15 (60%)		
History of neoplasms other than CRC	No	22 (47.8%)	24 (52.2%)	0.267	0.605
	Yes	3 (60%)	2 (40%)		
Imaging performed before or after the surgical intervention	After	6 (24%)	19 (76%)	12.284	0.000
	Before	19 (73.1%)	7 (26.9%)		
Nonsurgical therapy recieved	No	12 (75%)	4 (25%)	6.297	0.012
	Yes	13 (37.1%)	22 (62.9%)		
Outcome of the CRC	Alive with metastatic disease	8 (27.6%)	21 (72.4%)	12.368	0.002
	Alive without metastatic disease	14 (77.8%)	4 (22.2%)		
	Dead	3 (75%)	1 (25%)		
Final diagnosis of the patient	Metastatic colon cancer	11 (33.3%)	22 (66.7%)	9.206	0.002
	Non-metastatic colon cancer	14 (77.8%)	4 (22.2%)		

Table 4 provides an analysis of metastasis diagnosis mismatches in relation to the pathological and radiological characteristics of the primary lesion and the metastases. Regarding the TNM stage of the patient's colon cancer, there was no significant association observed between the stage and the metastasis diagnosis mismatch (X^2^ = 3.040, p = 0.386). Similarly, no significant association was found between the TNM stage of the primary tumor at the time of diagnosis and the metastasis diagnosis mismatch (X^2^ = 5.082, p = 0.166). However, the site of metastases showed a significant association with the metastasis diagnosis mismatch (X^2^ = 7.250, p = 0.027). Specifically, patients with liver metastases had a higher rate of mismatched diagnoses compared to those without liver metastases. The site of the primary tumor in the colon also demonstrated a significant association with the metastasis diagnosis mismatch (X^2^ = 18.435, p = 0.001). Mismatched diagnoses were more prevalent in patients with tumors located in the ascending colon and sigmoid colon while patients with tumors in the rectum had a higher rate of matched diagnoses. The histological type of the cancer did not show a statistically significant association with the metastasis diagnosis mismatch (X^2^ = 5.069, p = 0.079). Similarly, the differentiation grade of the tumor did not exhibit a significant association with the metastasis diagnosis mismatch (X^2^ = 4.026, p = 0.259). Finally, the size of the largest metastasis detected by MRI showed a significant association with the metastasis diagnosis mismatch (X^2^ = 15.909, p = 0.003). Mismatched diagnoses were more common when the metastasis size was larger (1-2 cm and greater than 3 cm) compared to smaller sizes (2-3 cm and less than 1 cm), as well as in cases where no metastasis was detected.

**Table 4:** Metastasis diagnosis mismatch in association with pathologic and radiologic characteristics of the primary lesion and the metastases (n=51).

Parameter		CT versus MRI metastasis diagnosis		X^2^	P-value
		Match	Mismatch		
TNM stage of the cancer	Stage I	1 (100%)	0 (0%)	3.040	0.386
	Stage II	2 (66.7%)	1 (33.3%)		
	Stage III	9 (60%)	6 (40%)		
	Stage IV	13 (40.6%)	19 (59.4%)		
Site of metastases	Liver	6 (30%)	14 (70%)	7.250	0.027
	None	12 (75%)	4 (25%)		
	Other	7 (46.7%)	8 (53.3%)		
Site of the primary tumor in the colon	Ascending colon	0 (0%)	5 (100%)	18.435	0.001
	Descending colon	4 (50%)	4 (50%)		
	Rectum	17 (81%)	4 (19%)		
	Sigmoid colon	3 (20%)	12 (80%)		
	Transverse colon	1 (50%)	1 (50%)		
TNM stage of primary tumor at the time of diagnosis	T1‎	1 (100%)	0 (0%)	5.082	0.166
	T2‎	0 (0%)	3 (100%)		
	T3‎	18 (56.3%)	14 (43.8%)		
	T4‎	6 (40%)	9 (60%)		
Histological type of colon cancer	Adenocarcinoma	24 (52.2%)	22 (47.8%)	5.069	0.079
	Mucinous adenocarcinoma	0 (0%)	4 (100%)		
	Signet ring cell carcinoma	1 (100%)	0 (0%)		
Differentiation grade of the tumor	Moderately differentiated	16 (44.4%)	20 (55.6%)	4.026	0.259
	Not specified	7 (70%)	3 (30%)		
	Poorly differentiated	1 (25%)	3 (75%)		
	Well differentiated	1 (100%)	0 (0%)		
Size of the largest metastasis detected by MRI	1-2 cm	5 (20.8%)	19 (79.2%)	15.909	0.003
	2-3 cm	3 (60%)	2 (40%)		
	Greater than 3 cm	1 (50%)	1 (50%)		
	Less than 1 cm	2 (100%)	0 (0%)		
	None	14 (77.8%)	4 (22.2%)		

The overall diagnostic accuracy of CT in detecting any metastases, compared to MRI as the reference standard, was estimated in Table 4, and the sensitivity was 87.8%, specificity was 77.8%, positive predictive value (PPV) was 87.8%, and negative predictive value (NPV) was 77.8%. For cases with no metastases, CT correctly identified none in all 13 cases, resulting in a specificity of 100%. Sensitivity and PPV could not be calculated as there were no TPs or FNs. The NPV was 100%. In cases with one metastasis, CT detected the presence of metastasis in five out of nine cases, yielding a sensitivity of 100%. The specificity was 75%, indicating that CT correctly identified the absence of metastasis in three out of four cases. The PPV was 83.33%, indicating the probability of a positive CT result accurately representing the presence of metastasis. The NPV was 100%. For cases with two metastases, CT correctly identified two metastases in four out of eight cases, resulting in a sensitivity of 66.67%. The specificity was 100%, indicating that CT accurately identified the absence of metastasis in all two cases without metastases. The PPV was 100%, indicating the probability of a positive CT result accurately representing the presence of metastasis. The NPV was 50%. In cases with three metastases, CT detected three metastases in five out of seven cases, yielding a sensitivity of 71.43%. Specificity could not be calculated as there were no TNs or FPs. The PPV was 100%, indicating the probability of a positive CT result accurately representing the presence of metastasis. The NPV was 0%. For cases with four or more metastases, CT correctly identified four or more metastases in 11 out of 14 cases, resulting in a sensitivity of 78.57%. Specificity could not be calculated as there were no TNs or FPs. The PPV was 100%, indicating the probability of a positive CT result accurately representing the presence of metastasis. The NPV was 0%.

## Discussion

The aim of this study was to compare the detection of metastases of CRC using CT and MRI in a single-center experience in Najran, Saudi Arabia. This study included 51 patients, and the results showed that both CT and MRI were effective in detecting metastases in patients with CRC, which is consistent with the literature [13-15]. The majority of patients had metastases measuring 1-2 cm, and the liver was the most common site of metastases. In terms of the number of metastatic lesions detected, both CT and MRI identified a range of lesions, with a similar proportion of cases having no detectable metastases.

The overall diagnostic accuracy of CT in detecting any metastases, compared to MRI as the reference standard, was found to have a sensitivity of 87.8%, a specificity of 77.8%, a PPV of 87.8%, and an NPV of 77.8%. These measures indicate the reliability of CT in correctly identifying the presence or absence of metastases. The high sensitivity suggests that CT is effective in detecting TP cases of metastases, while the specificity indicates its ability to correctly identify TN cases [16,17].

When analyzing specific scenarios, the diagnostic accuracy varied depending on the number of metastatic lesions. CT showed higher sensitivity in detecting a single metastasis (100%) compared to cases with two (66.67%) or three metastases (71.43%). However, the specificity was high in cases with two or more metastases, indicating that CT accurately identified the absence of metastasis in those cases.

This study also explored the association between patient characteristics, disease outcomes, and the presence of metastasis diagnosis mismatches between CT and MRI. Gender was found to be significantly associated with diagnosis mismatch, with a higher proportion of male patients experiencing a mismatch. The timing of imaging in relation to surgical intervention and the administration of nonsurgical therapy also showed significant associations with diagnosis mismatch. Additionally, disease outcome, as indicated by CRC outcome and the patient's final diagnosis, demonstrated significant associations with diagnosis mismatch.

Furthermore, the analysis of metastasis diagnosis mismatched with the pathological and radiological characteristics of the primary lesion, and the metastases revealed significant associations. The site of metastases and the site of the primary tumor in the colon showed significant associations with diagnosis mismatch. Mismatched diagnoses were more prevalent in patients with liver metastases and tumors located in the ascending colon and sigmoid colon.

The findings of this study align with comparable studies. The sensitivity and specificity values reported in this study are consistent with the existing literature [18-20], indicating that both imaging modalities are valuable tools in the evaluation of metastatic disease, with MRI being superior to CT.

Limitations of the study include potential selection bias due to a specific subset of patients with CRC being included, a small sample size which may impact the generalizability of the findings, variability in imaging techniques and expertise of radiologists, lack of consideration for timing of imaging and disease progression, and limited follow-up period. These limitations should be taken into account when interpreting the results of the study on the diagnostic accuracy of CT compared to MRI in detecting metastases in CRC patients. Further studies with larger sample sizes and multi-center designs are warranted to validate these findings.

## Conclusions

This study provides valuable insights into the detection of metastases of CRC using CT and MRI. Both imaging modalities demonstrated moderate sensitivity and specificity in detecting metastases, with variations depending on the number of lesions. The findings highlight the importance of considering patient characteristics, disease outcomes, and the pathological and radiological characteristics of the primary lesion and metastases in the interpretation of imaging results. CT and MRI play complementary roles in the diagnosis and management of CRC, and their combined use can improve the accuracy of metastatic disease detection.

## References

[1] Markowitz SD, Dawson DM, Willis J (20021). Focus on colon cancer. Cancer Cell.

[2] Benson AB 3rd, Bekaii-Saab T, Chan E (2013). Metastatic colon cancer, version 3.2013: featured updates to the NCCN Guidelines. J Natl Compr Canc Netw.

[3] Obaro AE, Burling DN, Plumb AA (2018). Colon cancer screening with CT colonography: logistics, cost-effectiveness, efficiency and progress. Br J Radiol.

[4] Nerad E, Lambregts DM, Kersten EL (2017). MRI for local staging of colon cancer: can MRI become the optimal staging modality for patients with colon cancer?. Dis Colon Rectum.

[5] Dighe S, Swift I, Brown G (2008). CT staging of colon cancer. Clin Radiol.

[6] Cascino TL, Leavengood JM, Kemeny N, Posner JB (1983). Brain metastases from colon cancer. J Neurooncol.

[7] Li Y, Eresen A, Shangguan J (2019). Establishment of a new non-invasive imaging prediction model for liver metastasis in colon cancer. Am J Cancer Res.

[8] Eresen A, Li Y, Yang J (2020). Preoperative assessment of lymph node metastasis in colon cancer patients using machine learning: a pilot study. Cancer Imaging.

[9] Taylor SA, Mallett S, Beare S (2019). Diagnostic accuracy of whole-body MRI versus standard imaging pathways for metastatic disease in newly diagnosed colorectal cancer: the prospective Streamline C trial. Lancet Gastroenterol Hepatol.

[10] Liu LH, Lv H, Wang ZC, Rao SX, Zeng MS (2019). Performance comparison between MRI and CT for local staging of sigmoid and descending colon cancer. Eur J Radiol.

[11] Earls JP (2000). Comparison studies of CT and MRI in patients with hepatic metastases. Oncology (Williston Park).

[12] Sica GT, Ji H, Ros PR (2000). CT and MR imaging of hepatic metastases. AJR Am J Roentgenol.

[13] Renzulli M, Clemente A, Ierardi AM (2020). Imaging of colorectal liver metastases: new developments and pending issues. Cancers (Basel).

[14] Granata V, Fusco R, de Lutio di Castelguidone E (2019). Diagnostic performance of gadoxetic acid-enhanced liver MRI versus multidetector CT in the assessment of colorectal liver metastases compared to hepatic resection. BMC Gastroenterol.

[15] Kijima S, Sasaki T, Nagata K, Utano K, Lefor AT, Sugimoto H (2014). Preoperative evaluation of colorectal cancer using CT colonography, MRI, and PET/CT. World J Gastroenterol.

[16] Kwak JY, Kim JS, Kim HJ, Ha HK, Yu CS, Kim JC (2012). Diagnostic value of FDG-PET/CT for lymph node metastasis of colorectal cancer. World J Surg.

[17] Filippone A, Ambrosini R, Fuschi M, Marinelli T, Genovesi D, Bonomo L (2004). Preoperative T and N staging of colorectal cancer: accuracy of contrast-enhanced multi-detector row CT colonography--initial experience. Radiology.

[18] Potter KC, Husband JE, Houghton SL, Thomas K, Brown G (2009). Diagnostic accuracy of serial CT/magnetic resonance imaging review vs. positron emission tomography/CT in colorectal cancer patients with suspected and known recurrence. Dis Colon Rectum.

[19] Sivesgaard K, Larsen LP, Sørensen M (2018). Diagnostic accuracy of CE-CT, MRI and FDG PET/CT for detecting colorectal cancer liver metastases in patients considered eligible for hepatic resection and/or local ablation. Eur Radiol.

[20] Brendle C, Schwenzer NF, Rempp H (2016). Assessment of metastatic colorectal cancer with hybrid imaging: comparison of reading performance using different combinations of anatomical and functional imaging techniques in PET/MRI and PET/CT in a short case series. Eur J Nucl Med Mol Imaging.

